# The effectiveness of demand creation interventions for voluntary male medical circumcision for HIV prevention in sub‐Saharan Africa: a mixed methods systematic review

**DOI:** 10.1002/jia2.25299

**Published:** 2019-07-22

**Authors:** Samuel Ensor, Bethan Davies, Tanvi Rai, Helen Ward

**Affiliations:** ^1^ School of Public Health Imperial College London London United Kingdom

**Keywords:** HIV infections, Circumcision; Male, Systematic review, Programme evaluation, Africa South of the Sahara, Health Services Needs and Demand

## Abstract

**Introduction:**

UNAIDS has recommended that in 14 countries across sub‐Saharan Africa (SSA), 90% of men aged 10 to 29 years should be circumcised by 2021 to help reduce transmission of HIV. To achieve this target demand creation programmes have been widely implemented to increase demand for Voluntary Medical Male Circumcision (VMMC). This review explores the effectiveness of demand creation interventions and factors affecting programme implementation.

**Methods:**

We completed a mixed methods systematic review searching Medline, Embase, Global health, psycINFO and CINAHL databases in August 2018 with no time restrictions. Demand creation interventions conducted in SSA were categorized and quantitative data about VMMC uptake was used to compare relative and absolute effectiveness of interventions. Qualitative data were summarized into themes relevant to the delivery and impact of programmes.

**Results and discussion:**

Eighteen of the 904 titles were included in the review. Effective interventions were identified in each demand creation category: financial incentives, counselling or education, involvement of influencers and novel information delivery. Of the 11 randomized controlled trials (RCTs), the greatest absolute impact on VMMC prevalence was seen with a complex intervention including VMMC promotion training for religious leaders (compared to control: 23% (95% CI 22.8 to 23.8) absolute increase; odds ratio (OR) 3.2 (1.4 to 7.3)). Financial incentives generally produced the largest relative effects with men up to seven‐times more likely to undergo VMMC in the intervention arm compared to control (adjusted OR 7.1 (95% CI 2.4 to 20.8), 7.1% (3.7 to 10.5) absolute increase). Qualitative findings suggest that interventions are more impactful when they are judged appropriate and acceptable by the target population; delivered by people with relevant personal experience; and addressing broader social and cultural influences through partnership with and education of community leaders.

**Conclusions:**

A range of demand creation interventions can increase VMMC uptake. The most acceptable and effective interventions are financial incentives framed as fair compensation (relative effect) and programmes of education or counselling delivered by people who are influential in the community (absolute effect). Future research should include larger studies with longer follow‐up and a consistent definition of VMMC uptake.

## 1 Introduction

Despite global Human Immunodeficiency Virus (HIV) incidence peaking in 1997, prevalence continues to rise 1. There are an estimated 36.9 million people currently infected, with 53% residing in sub‐Saharan Africa (SSA) 1.

Voluntary medical male circumcision (VMMC) is 50% to 60% effective at preventing the acquisition of HIV infection in men 2, 3, 4. UNAIDS had an original target to reach 80% population coverage in men aged 15 to 49 years, in order to avert an estimated 6 million infections and 3 million deaths by 2025 in priority countries 5. This target has now been increased to 90% coverage in men aged 10 to 29 years 6. By 2017, over 90% of the 20.8 million target number of VMMCs had been performed, averting an estimated 230,000 HIV infections 6. Advances in technology and increased VMMC availability have contributed to recent increases in the uptake of the procedure, but uptake remains limited by poor demand in some target countries 7.

The HIV Prevention Cascade has been suggested as a framework that maps the steps required to prevent transmission 8, 9, 10. It is broadly based around three domains: demand, supply and adherence, or more recently refined as motivation, access and use 11. It is intended to help identify issues surrounding poor adoption of HIV prevention in different populations and for specific technologies. When applied to VMMC, the key elements of the cascade are demand, or motivation and access. Once VMMC is carried out there is no ongoing issue with adherence, although some cascades include a final step of efficacy 10.

Demand creation programmes have been implemented in many settings to encourage VMMC uptake 7, 12; however until recently the evidence for the effectiveness of these interventions has been limited. In 2016, an eleven‐paper series assessed the effectiveness of seven different VMMC demand creation programmes in SSA. Sgaier *et al*. reviewed the impact evaluations from five randomized controlled trials (RCTs) and two quasi‐experimental studies 13, 14, 15, 16, 17, 18, 19, 20, 21, 22. Three interventions, two offering financial incentives 19, 20 and one novel football‐based education sessions 16, reported significant impact, while qualitative components from others gave good insight into their implementational shortcomings 7. Two further systematic reviews report the beneficial impact of financial incentives on VMMC uptake 23, 24. The meta‐analysis of six financial interventions reported that men in the intervention arm were almost five times more likely to undergo the procedure compared to the control arm (combined odds ratio (OR) 4.78 (95% CI 4.17 to 5.48)) 23. Another recent systematic review has summarized the overarching barriers and facilitators to VMMC uptake by men in SSA 25. The authors identified important differences in the key factors acting at the community, service provider and individual/interpersonal level across priority settings.

To date, systematic reviews have focused on the quantitative impact of financial incentives. This review aims to assess the effectiveness of all VMMC demand creation interventions at increasing VMMC uptake and to synthesize the factors facilitating and impeding effective implementation from the perspective of the demand creation component of the HIV Prevention Cascade.

## 2 Methods

### 2.1 Selection criteria

A mixed methods systematic review was conducted. The full inclusion criteria are presented in Appendix S1. Briefly, study populations must include males older than 10 years, matching the age used in a previous systematic review 12, and studies must have taken place in SSA. Study designs eligible for inclusion were RCTs, quasi‐experimental, case‐control, cohort, comparative and observational. Conference abstracts, letters or editorials were excluded, and no time or language restrictions were imposed.

### 2.2 Search strategy

Searches were conducted in Medline, Embase, Global Health, psycINFO and CINAHL using common search strategies consisting of keywords and MeSH during March and April 2017 and updated in August 2018. The search strategy was developed with the assistance of a Medical Librarian (Appendix S2). Search terms were based upon those used in Cochrane reviews and were structured around five concepts; VMMC (e.g. “Circumcision,” “VMMC”), HIV (e.g. “hiv,” “human immunodeficiency virus”), Outcome (e.g. “uptake,” “demand*”), Location (SSA) and study design (e.g. “randomi*,” “comparative*”) 26, 27. Database searching was supplemented with reference list searching, including of the previously published reviews, and monitoring of published literature (e.g. with Google Scholar).

### 2.3 Data analysis

Full titles and abstracts of the search results were compiled in reference management software. Duplicates were removed, and the remaining results had pre‐defined inclusion and exclusion criteria (Appendix S3) applied to them by two reviewers (BD & SE). Discrepancies in the articles deemed fit for inclusion were settled by consensus.

Studies that met the inclusion criteria were assessed for quality by one reviewer (SE), using the Mixed‐Methods Appraisal Tool (MMAT), with queries being settled by consensus within the review team. An *a priori* cut off > 25% was chosen for studies to be eligible for inclusion. All included studies were reviewed.

One reviewer (SE) extracted and a second (BD – quantitative; HW – qualitative) checked data from the included articles using a template: setting, study design and methodology, sample (intervention population and sample sizes), blinding and randomization methods, intervention, control, duration, quantitative and qualitative outcomes (primary and secondary), limitations and funding. Demand creation interventions were reviewed and categorized into broad approaches. For the quantitative analysis, descriptive data including study setting, population and intervention duration were compared, individual intervention effect sizes (ORs) were analysed and any change in VMMC uptake was compared. We calculated the absolute impact of intervention on the outcome, *post* hoc, where the available data was included in the study. Qualitative studies were reviewed and three authors (SE, TR, HW) extracted details of aims, methods, outcome measures, key findings and conclusions. These were then summarized into themes relevant to the delivery and impact of demand creation intervention programmes.

## 3 Results

### 3.1 Search results

**Figure 1:**
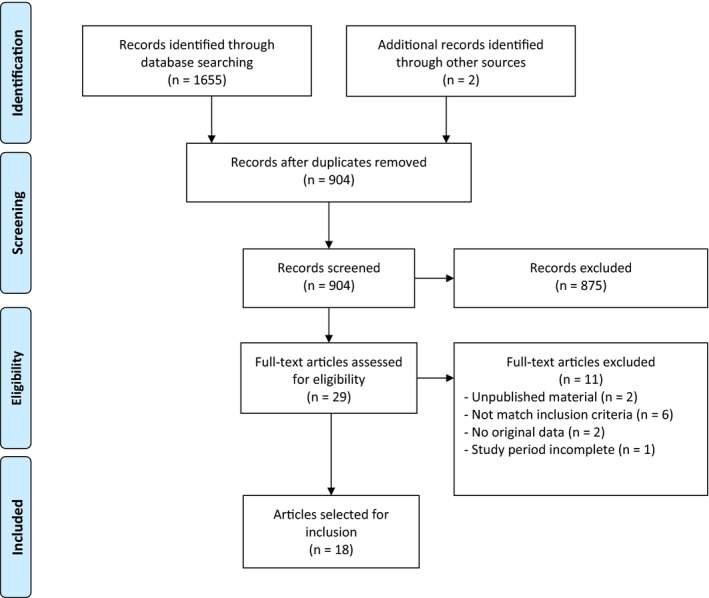
PRISMA flowchart.

A total of 1655 articles were returned (Figure 1). Reference list searching identified two further articles. Duplicates (n = 754) were removed and 903 papers underwent title and abstract screening. Full text was obtained for 29 titles; 18 were included in the final review.

### 3.2 Study characteristics

The 18 papers included were published between 2014 and 2018 and presented data associated with 16 studies; five cluster randomised control trials (CRCTs) 13, 14, 16, 28, 29, 30, six RCTs 17, 19, 20, 31, 32, 33, two prospective interventional cohorts 34, 35, two quasi‐experimental 18, 36 and one “non‐experimental” study (amended from an RCT due to delays with VMMC service implementation) 21 (Table 1). Studies took place across seven countries in SSA. Most of the studies (n = 14) had single country settings; South Africa (n = 3), Zambia (n = 3), Tanzania (n = 2), Kenya (n = 2), Uganda (n = 2), Zimbabwe (n = 1) and Malawi (n = 1), while 1 study took place in both South Africa and Uganda. The majority (n = 12) of the included papers presented only quantitative data (Table 2), three were only qualitative, obtained through in‐depth interviews (IDIs) and focus group discussions (FGDs), and three presented both quantitative and qualitative results. The size of the study populations varied greatly across studies: quantitative study populations ranged from 522 to 145,028 participants and qualitative study populations ranged from 10 to 289.

**Table 1:** Summary of studies included in analysis

Author (date)	Setting (VMMC prevalence)	Population	Study date (F/U months)	Sample size		Study design
				Quant	Qual	
Barnabus (2016)	Rural South Africa & Uganda	Uncircumcised HIV‐negative men aged 16 to 49, with private text‐messaging	Jun 2013 to Mar 2015 (9)	750	‐	RCT
Bazant (2016)	Rural Tanzania (49% to 60%)	Uncircumcised men aged >20	Nov 2014 to Feb 2015 (3)	1186	72	Cluster randomized evaluation + FGD
Cook (2016)^a^	Urban Zambia	Sub‐group: uncircumcised, HIV negative men aged >18 with female partner	Not presented	668	‐	CRCT
DeCelles (2016)^b^	Urban Zimbabwe	Men aged 18 to 30 playing in football clubs and trial coaches	2012 to 2013	‐	46	IDIs + FGD
Downs (2017)	Rural Tanzania (<20%)	Total male population	Jun 2014 to Dec 2015 (7)	145,028	‐	CRCT + FDG
Evens (2016)^c^	Rural & Urban Kenya	Thirumurthy (2014) trial participants	Not presented	‐	64	IDI
Kaufman (2016)^b^	Urban Zimbabwe (>20%)	Male students aged 14 to 20	Mar to Oct 2014 (4)	1148	‐	CRCT
Leiby (2016)	Urban & peri‐urban Zambia	Uncircumcised male subscribers to national SMS platform aged 15 to 30	May to Oct 2014 (6)	1652	‐	RCT
Marshall (2017)	Peri‐urban South Africa (57%)	Uncircumcised men	Aug to Nov 2015 (2)	226	‐	Prospective cohort
Montague (2014)	Rural South Africa	HIV‐negative male students aged 11 to 20 at 42 selected high schools	Mar 2011 to Feb 2013 (24)	11,088	‐	Prospective cohort
Miiro (2017)	Peri‐urban Uganda	Uncircumcised male students in Forms 2 and 3 (median age 16 to 17)	Oct to Nov 2015	‐	10	IDI
Semeere (2016)	Urban Uganda (28%)	Pregnant women with an uncircumcised spouse	May 2014 to Jan 2015 (3)	601	117	Retrospective pre/post study +IDI
Thirumurthy (2014)^c^	Urban & Rural Kenya	Uncircumcised men aged 25 to 49	Jun 2013 to Feb 2014 (2)	1502	‐	RCT
Thirumurthy (2016)	Urban & Rural Kenya (< 80%)	Uncircumcised men aged 21 to 39	Apr to Sept 2014 (3)	911	‐	RCT
Thornton (2016)	Urban Malawi (~2%)	Uncircumcised men aged 18 to 30	2010 (3)	1649	‐	RCT
Weiss (2015)^a^	Urban Zambia (12%)	Uncircumcised, HIV‐negative men aged >18 with female partners (optional)	Feb 2012 to Oct 2014 (12)	800	‐	CRCT
Wilson (2016)	Peri‐urban South Africa (25.2%)	Men aged >18	Jun to Aug 2014 (2)	4000	‐	RCT + FDG
Zanolini (2016)	Rural Zambia	Men aged ≥18	Jun 2014 to Feb 2015 (5)	N/A	‐	Time series (amended RCT)

ART, anti‐retroviral therapy; CRCT, Cluster randomized control trial; F/U, follow‐up; FGD, focus group discussion; IDI, individual in‐depth interviews; MTC, “Make the cut;” Quant, quantitative; Qual, qualitative; RCT, randomized control trial; VMMC, voluntary medical male circumcision.

^a^Contain data from same study; ^b^both contain data from MTC+ study. DeCelles (2016) also contains data from separate MTC study; ^c^contain data from same study.

**Table 2:** Quantitative study design and results

Author	Intervention	Control/comparator	Outcome measure	Descriptive data	Absolute change in VMMC uptake (intervention minus control)	Relative change/Effect size
Barnabus	Standard of care + randomized to mobile phone SMS reminders or home visits to promote service linkage	Counselling about VMMC, referral card to local circumcision facilities	VMMC uptake (three months)	Control: 62/224 (27%) SMS: 137/284 (48%) Counsellor: 106/226 (47%)	**20.6% (12.3 to 28.8)19.2% (10.5 to 28.0)**	**RR 1.7 (1.4 to 2.2)RR 1.7 (1.3 to 2.1)**
Bazant	Control + invite to weekly lottery (for smartphone worth $85.60) after VMMC	Mass media messages and peer‐promotor conversations	Change in number of VMMCs compared to previous year	Control 8% ↑ (pre n = 257, post n = 278) Intervention 47% ↑ (pre n = 264, post n = 388)	39% ↑ (NS)	Not presented
Cook	Spear and shield: for men see Weiss (2015). Female partners received four separate weekly group education sessions + $6/visit	For men see Weiss (2015). Female partners: four video‐based health education group sessions + $6/visit	Likelihood of VMMC uptake	Control: 69/328 (20.9%) Intervention:141/340 (41.4%)	**20.4% (13.6 to 27.3)**	**OR** a **2.7 (1.9 to 3.7)**
Downs	Day of training for church leaders + follow up group or individual discussions every two weeks	Standard outreach	% of population attending for VMMC	Control: 25,484/86,492 (29.5%) Intervention: 30,889/58,536 (52.8%)	**23.3% (22.8 to 23.8)**	**OR 3.2 (1.4 to 7.3)**
Kaufman	“MTC+”: football‐based group education session (one hour) by trained facilitator + contact for further group meetings + transport to VMMC + non‐monetary incentive worth $5	Usual care	% VMMC uptake in uncircumcised at baseline	Control: 17/371 (4.6%) Intervention: 37/304 (12.2%)	**7.6% (3.4 to 12.1)**	**OR 2.7 (1.2 to 5.9)**
Leiby	Conventional or tailored set of 21 SMS test messages about VMMC	Routine access to SMS service to engage counsellors on any topic	Self‐reported VMMC uptake	Control: 57/771 (7.4%) Conventional: 66/770 (8.6%) Tailored: 67/771 (8.7%)	1.2% (−1.5 to 3.9) 1.3% (−1.4 to 4.0)	AOR^b^ 1.2 (0.8 to 1.7) AOR^b^ 1.2 (0.8 to 1.8)
Marshall	Three individual motivational interviews and $17 post‐VMMC	Baseline circumcision prevalence	Circumcision prevalence	Baseline: 296/522 (56.7%, (52.4% to 60.9%)) Final:425/522 (81.4%. [77.9% to 84.6%)])	**24.7% (19.3 to 30.1)** ***p *** **<** *** *** **0.001**	
Montague	Community engagement + in‐school VMMC awareness sessions + peer recruitment + travel to clinic	70% prevalence target	# of VMMCs performed	5165/11,088 (47% prevalence)	No baseline data presented	
Semeere	Education on VMMC + communication skills training for women + transport voucher ($8.50) redeemed after VMMC	Standard care + $8.50 transport voucher for men undergoing VMMC	% of women whose spouse had VMMC by one month	Control: 4/296 (1.4%) Intervention: 7/305 (2.3%)	0.9% (−1.2 to 3.1)	OR 1.5 (0.4 to 5.2)
Thirumurthy (2014)	Food vouchers for $2.50, $8.75 or $15 after VMMC	Information about nine clinics providing free VMMC	% VMMC uptake	Control: 6/370 (1.6%) $2.50: 7/376 (1.9%) $8.75: 25/381 (6.6%) $15: 34/377 (9.0%)	0.3% (−1.6 to 2.1) **4.9% (2.1 to 7.7)7.4% (4.2 to 10.6)**	AOR^c^ 1.1 (0.4 to 3.3) **AOR** c **4.3 (1.7 to 10.7)AOR** c **6.2 (2.6 to 15.0)**
Thirumurthy (2016)	Food voucher for $12.50 or entry into lottery (expected values $12.50) after VMMC	Information about free VMMC clinics + $0.6 voucher after VMMC	% VMMC uptake	Control: 4/299 (1.3%) Voucher: 26/308 (8.4%) Lottery: 10/302 (3.3%)	**7.1% (3.7 to 10.5)** 2.0% (−0.4 to 4.4)	**AOR** c **7.1 (2.4 to 20.8)** AOR^c^ 2.5 (0.8 to 8.1)
Thornton	Voucher subsidized VMMC (cost $0‐$6)	Free VMMC	% VMMC uptake	>$0: 30/1257 (2.39%) $0: 12/392 (3.0%)	0.7% (−1.2 to 2.6)	OR^a^ 1.3 (0.7 to 2.6)
Weiss	“Spear and shield”:4 weekly 90‐ minute group education sessions + $6 per assessment	4 weekly 90‐minute video‐based group education sessions on endemic diseases + $6	Likelihood of VMMC uptake	Control: 96/400 (24%) Intervention: 161/400 (40%)	**16.3% (9.9 to 22.6)**	**AOR** d **2.5 (1.2 to 4.9)**
Wilson	Control postcards + offer of $10 to attend for counselling, challenge message or novel VMMC information	Postcards with routine VMMC information + clinic details	# of VMMCs	Overall 74/4000 men returned postcards $10:?/1000 Challenge:?/1000 Information:?/1000		**OR 5.3 (2.2 to 12.8)OR 2.7 (1.1 to 6.9)** OR 1.7 (0.6 to 4.6)
Zanolini (2016)	Clients undergoing VMMC asked to refer ≤5 uncircumcised men, paid $2/referral	Comparison with 2012 health data rends trends from non‐intervention facilities	Mean monthly difference in # of VMMCs	Control: not presented Active Intervention: 848 VMMCs, 2402 (699 men given vouchers; 348 used vouchers)	Mean monthly difference 7.6 VMMCs (−20.4 to 40.8)	Adjusted mean monthly difference 10.2 (−18.3 to 33.9)

Statistically significant effect sizes are shown in bold.AOR, Adjusted odds ratio; CRCT, cluster randomized control trial; M, men; MTC, “Make the Cut;” F/U, follow‐up; NS, Non‐significant; OR, Odds ratio; RCT, randomized control trial; RR, Relative risk; VMMC, Voluntary medical male circumcision; W, women.

^a^OR and CIs calculated by SE/BD; ^b^adjusted for intention level, adulthood, district, circumcised family members, high‐uptake tribe, number of surveys to which individual responded, and verifiability. effect size calculated after loss to follow up; ^c^adjusted for age, educational attainment, marital status and wealth; ^d^adjusted for age, education level and baseline stage of readiness for voluntary medical male circumcision.

### 3.3 Quality assessment

No major limitations or conflicts of interest were found during data extraction. All eligible studies received at least 25% on MMAT and were included in the analysis (Appendix S4).

### 3.4 Quantitative results

Interventions were categorized into four major types: (i) financial incentives, (ii) counselling or education for prospective candidates, (iii) education and involvement of influencers, and (iv) novel information delivery, although some included more than one of these approaches. Most (n = 13) studies 13, 16, 17, 18, 19, 20, 21, 28, 29, 31, 32, 33, 34 presented data on the number of circumcisions completed at the end of the study period, while the remaining two studies 30, 35 reported the prevalence of circumcision in the cohort. Table 2 shows the key findings from each study with both relative and absolute effects (where reported in the paper or calculated from raw data).

#### 3.4.1 Financial incentives

Six of the studies in this review describe interventions with a financial incentive component not offered to the control or comparator groups 13, 19, 20, 31, 32, 34. Overall, financial incentives worth at least two‐days wages – food vouchers (over $8.75) (n = 2) or conditional cash transfers (>$10) (n = 1) – were found to have a large significant effect on the likelihood of having a VMMC procedure (AOR 4.3 to 7.1) but a small absolute change in VMMC uptake compared to the control group (<7.5%) 19, 20, 31. A $17 conditional‐cash‐transfer combined with motivational interviews was associated with a 25% increase in VMMC uptake in an observational cohort 34. Lottery‐based incentives (n = 2) did not demonstrate an impact on the number of VMMCs performed in the intervention compared to control groups 13, 19.

Thirumurthy *et al*. conducted two RCTs to explore the effect of giving different valued food vouchers on VMMC uptake (Table 2) 19, 31. Vouchers were equivalent to transport costs plus one to three days wages. Vouchers worth US$15 (adjusted OR (AOR) 6.2 (2.6 to 15.0)) 31, $12.50 (7.1 (2.4 to 20.8)) 19 and $8.75 (4.3 (1.7 to 10.7)) significantly increased VMMC uptake 31. The $15 compensation was not significantly more effective than $8.75 (*p *=* *0.21) 31. The authors also found that the interventions were effective at increasing the likelihood of VMMC uptake in traditionally harder to reach groups, including married men (AOR 4.5 (2.3 to 9.1)) and men over 33 years (AOR 7.9 (2.7 to 22.7)) 31. Wilson *et al*. conducted a RCT comparing a $10 conditional‐cash‐transfer advertised via postcards containing information about the benefit of VMMC 20. The intervention group had a large relative increase in VMMC uptake compared to the control group (OR 5.30 (2.20 to 12.76)) but the absolute effect was not presented. The study was markedly underpowered (74 out of 4000 postcards returned compared to an estimated sample size of 2484).

Bazant *et al*. randomized pairs of facilities into providing VMMC alone or VMMC plus entrance into a lottery for a smart‐phone 13. There was a non‐significant increase in the absolute number of VMMCs performed in both the intervention and control facilities compared to the number performed in the same facilities during the previous 12 months (47% (*p *=* *0.150) and 8% (*p *=* *0.850) respectively) 13. The RCT by Thirumurthy *et al*. also included a lottery incentive with an expected value of $12.50 (equivalent to the food voucher). Participants in the lottery arm were not significantly more likely to have a VMMC compared to the control group (AOR 2.5 (0.8 to 8.1)) 19. Thornton *et al*. studied the effect of offering subsidized circumcision (at different rates) on VMMC uptake 32. Discounting the cost to $0 did not lead to a significant increase in VMMC uptake compared to the men paying between $0.55 and $6.75 for the procedure (OR 1.3 (0.7 to 2.6)). There was no absolute difference in uptake between the $0 group and the others (3.06% (n = 12/392) compared to 2.39% (n = 30/1257), *p *=* *0.4597). This study looked at a secondary outcome of VMMC uptake by voucher value and *ex ante* sexual risk which suggests that men with the highest sexual risk behaviours only underwent VMMC when it was offered for free.

The study by Marshall *et al*. describes the change in VMMC prevalence in the study population offered a nine‐week programme of individual motivational interviews plus $17 post‐VMMC 34. The prospective cohort did not have a control arm and both the counselling and cash transfer were given to all men who underwent VMMC. 522 men were invited to participate in the cohort, of whom 226 were uncircumcised and 148 (69.8% (63.4 to 75.7)) presented for VMMC within nine weeks. Nine procedures were delayed for medical reasons and the authors estimate that during follow‐up circumcision prevalence rose significantly from 56.7% (52.4 to 60.9, 296/522) to 81.4% (77.9 to 84.6, 425/522) (*p *<* *0.001), a 24.7% absolute increase 34.

#### 3.4.2 Counselling or education

Four included studies considered the impact of group‐education or awareness raising interventions on VMMC uptake 16, 28, 29, 35. A further two studied the effect of individual counselling on participation 33, 34. Overall, group education sessions increased the likelihood of VMMC around 2.5‐fold with a 8% to 20% absolute increase in uptake compared to control groups 16, 28, 29. It is estimated that 13 uncircumcised men need to participate with group education for one to undergo VMMC 16. Individual‐level counselling was shown to increase the uptake of VMMC by men who attended a circumcision facility by almost 70% compared to the control group (absolute increase 19%) 33.

The study by Marshall described in the preceding section is not considered further here as it is not possible to disentangle the effect of the motivational interviewing from the conditional cash transfer 34. Barnabus *et al*. undertook an RCT comparing the effectiveness of two interventions: SMS reminders and lay‐counsellor follow‐up, on VMMC uptake in men who participated with HIV testing and attended a circumcision facility 33. The men who received lay‐counsellor follow‐up after their visit to the circumcision facility were almost 70% more likely to undergo VMMC than the men randomized to receive promotion materials (RR 1.67 (1.29 to 2.14), absolute increase 19%) 33.

Weiss *et al*. undertook a CRCT of the “Spear and Shield” intervention, which compared the uptake of VMMC in men participating in specific HIV‐focused group education sessions to men participating with control education sessions about endemic diseases 28. Participants in both arms were given $6 reimbursement per assessment. When compared to the control group, men in the intervention arm were 2.5 times more likely to undergo VMMC (AOR 2.45 (1.24 to 4.90)), an absolute increase of 16.3% (9.9 to 22.6) 28. Men were encouraged to enrol in the study with their female partners. For those who did, Cook *et al*. conducted a sub‐group analysis (n = 668/800) which identified a similar absolute increase in uptake of VMMC in the intervention compared to control group (20.4% (13.6 to 27.3)) 29. The authors estimated that an additional 5.9% of men received VMMC due to positive changes in their partner's attitudes.

Kaufman *et al*. undertook a CRCT of the “MTC+ (Make‐The‐Cut‐Plus)” football‐based group education intervention, for male students aged 14 to 20 years 16. The intervention was delivered by trained “coaches” who followed‐up and supported participants interested in becoming circumcised. Of adolescents who were uncircumcised at baseline, those in the intervention schools were significantly more likely to undergo VMMC compared to those in the control schools (OR 2.65 (1.19 to 5.86)) 16. Montague *et al*. describes a further school‐based intervention that used a phased approach including community engagement, VMMC awareness sessions and peer‐recruitment 35. Peer‐recruiters were early adopters of VMMC and were given vouchers worth $3 for cell‐phones plus other small prizes (t‐shirts, watches etc.). The intervention included a competition element with prizes for recruiters and schools. Of the 11,088 male students at the 47 selected schools, 47% (n = 5165) underwent a circumcision during the two‐year intervention. No data is presented on baseline circumcision rates and there is no comparator group.

#### 3.4.3 Influencers: education and involvement

Four studies trained “influencers” (religious leaders (n = 1), female partners (n = 1), circumcised peers (n = 2)) to promote VMMC within their partnerships, community or social circle 18, 21, 30, 35. Training Church leaders was associated with a 23% increase in the proportion of the population attending for VMMC compared to control settings and was one of the few studies to show impact at scale 30. In contrast, training female partners and peers has not been shown to increase VMMC uptake.

The study by Montague *et al*. has been considered in the earlier section as the peer‐recruiter role formed part of a complex intervention with an educational component 35. Downs *et al*. conducted a CRCT which trained church leaders to educate and promote VMMC to their congregations (one‐day course) 30. Eight pairs of villages were randomized with an estimated population of 145,028 exposed to Church leaders’ teachings. In the eight intervention villages 1194 leaders received training. The proportion of the male population who had presented for VMMC by the end of the study was 23.3% (22.8 to 23.8) higher in intervention villages compared to the paired control villages (52.8% compared to 29.5%; OR 3.2 (1.4‐7.3)) The authors estimated that this led to an additional 13,000 circumcisions being performed in the intervention villages 30.

Peer‐recruiters were the sole intervention in the study by Zanolini *et al*. The authors offered men who had recently undergone circumcision small monetary incentives ($2 per referee) to promote it to their peers 21. The majority of eligible men (82%) enrolled in the intervention but the mean number of circumcisions performed per month did not significantly increase compared to the comparator non‐intervention facilities (change in mean monthly VMMCs 7.6 (−20.37 to 40.83)) 21.

Semeere *et al*. undertook a pre/post comparison of VMMC rates in the partners of pregnant women to evaluate the impact of educating pregnant women about VMMC 18. Absolute VMMC uptake was very low in both periods (intervention 7/305 and control 4/296) compared to *a priori* estimates and no effect on VMMC uptake in the month following the intervention was seen (OR 1.5 (0.4 to 5.2)).

#### 3.4.4 Novel information delivery

Three studies included in this review compared the impact of SMS text message packages (n = 2) or innovative postcard messages (n = 1) on the uptake of VMMC 17, 20, 33. Delivering messages that were challenging or encouraging were shown to significantly increase VMMC uptake over the control groups.

The RCT by Barnabus *et al*. described above also included an SMS “encouragement” arm where men who attended a circumcision facility were sent a message (“this could be the best decision you make – act now!”) three weeks after their HIV test and a phone‐call at four weeks 33. The men in the SMS arm were 70% more likely to undergo VMMC than those randomized to receive promotion materials (RR 1.72 (1.36 to 2.17), absolute increase 20%) 33.

Leiby *et al*. undertook an RCT comparing the impact of packages of informative and motivational text messages, either tailored or untailored to the individual's self‐reported stage of behavioural change 17. Text message packages were found to increase the proportion contacting a VMMC counsellor for more information compared to the control (53%, n = 817/1541 compared to 19%, n = 143/771) however neither conventional (AOR 1.17 (0.80 to 1.72)) nor tailored (AOR 1.24 (0.84 to 1.81)) packages demonstrated a significant effect on self‐reported VMMC uptake 17.

Wilson *et al*. compared the impact of postcards with a message “challenging” men to become circumcised (“Are you tough enough?”) or a novel VMMC health benefit to control postcards containing basic VMMC and clinic information 20. The “challenge” postcards were associated with a significant increase in VMMC uptake at two months (OR 2.69 (1.05 to 6.91)) compared to control, but those displaying information only did not demonstrate a significant difference from the control group (OR 1.67 (0.61 to 4.62)) 20.

### 3.5 Qualitative results

The qualitative studies explored a range of outcomes including preferences, acceptability, attitudes and perceptions of programme components and delivery. Participants in interviews and focus groups included men who were and were not circumcised, their female partners, and key informants involved in implementation. The details of each study including key findings are summarized in Table 3 13, 14, 15, 18, 30, 36.

**Table 3:** Qualitative study designs and results

Author	Study design	Intervention	Main aim and outcomes for qualitative study	Key findings from qualitative study	Authors’ conclusion	Strengths and limitations
Bazant (2016)	Focus groups with sub‐set of clients who had undergone circumcision and peer educators as part of randomized evaluation of lottery	Entry into weekly lottery for smartphone worth $85.60 conditional on becoming circumcised	Preferences for VMMC incentives	The lottery created interest in VMMC – “a buzz” Suspicion about the phone incentive which was too high in value Preference for an incentive for all (rather than lottery) Mixed views, but preference for money as reimbursement (rather than phone)	The lottery might work at some stages of a programme, e.g. late adopters, but not when need wide uptake	Little detail on method to assess quality, e.g. what was asked in focus groups. No data from those who did not seek VMMC
Evens (2016)	In depth interviews with circumcised and uncircumcised men and female partners as part of RCT of financial compensation. Inductive thematic analysis	Food vouchers of varying amounts conditional on becoming circumcised	Perceptions of how compensation provision influenced decisions about circumcision	Loss of income is a significant barrier to circumcision and the financial compensation programme helped motivate men in three ways: (a) removed the financial barrier, without the money they would not have been circumcised; (b) the money prompted it in men who had already decided to be circumcise, i.e. it was a “nudge;” (c) it was the information that prompted them, the money was a bonus only Those who did not get circumcised cited (a) the compensation was insufficient; (b) a primary reason other than finance (e.g. not discussed it with female partner, fear of pain); (c) they felt the decision should not be linked to compensation Female partners were supportive of decisions, but thought the compensation was insufficient There was no evidence that economic compensation was perceived as being coercive	Financial compensation can be an important tool in increasing circumcision uptake, but the amount needs to be carefully judged, and other barriers, notably fear of pain, also need to be addressed	Relatively small sample size but included both circumcised and uncircumcised men and their female partners. Methods are clearly described
DeCelles (2016)	A process evaluation with in depth interviews and focus groups with soccer coaches, circumcised and uncircumcised men linked to RCT	Soccer‐themed educational session and follow‐up to promote circumcision	Perceptions of programme impact, intervention components and delivery; understanding of intervention content; factors related to uptake	Coaches’ individual stories were helpful in sharing knowledge about circumcision and in motivating boys and men, with the coach‐participant relationship being particularly valued and trusted Older men were less likely to be convinced that it was relevant to them Follow‐up texts and coaches accompanying participants to the clinic were highly valued by some	The programme was acceptable, the quality of the coach‐participant relationship was highly valued, particularly discussion of personal experience	Small sample size particularly in MTC
Miiro (2017)	Mixed methods: cross sectional survey and in‐depth interviews with male school students associated with feasibility study in Uganda	Soccer‐themed educational session and follow‐up to promote circumcision	Acceptability, feasibility and perceptions of implementation of a soccer‐based intervention among schoolboys	General favourable towards circumcision and good acceptability in principle Feasibility study showed need for further engagement with parents and school to improve uptake Interviews showed importance of family and peer support in preparing participants for circumcision Sessions with the coaches were found to prompt decision in those who were already receptive, helped by their personal experience of the procedure and individual follow‐up after The main reasons for getting circumcised were hygiene and reduced HIV risk, while main reasons against were fear of pain, loss of contact with the coach or family opposition	The intervention can be adapted and effective but needs to attend to the key role of family and peer support, and to address practical issues of timing and delivery through schools	A small feasibility study, but able to identify some key factors that could help improve implementation of MTC in a new setting
Semeere (2016)	Nested interview study as part of quasi‐experimental behaviour change intervention study. Interviews with women, men and key informants.	Education for pregnant women to encourage them to refer their male partners for circumcision	Evaluation of the causal chain of the intervention including women's perceptions of benefits of circumcisions, and how the information may have affected men's decisions	After the intervention, women had a high level of discomfort about talking to their partners about circumcision but they mostly still delivered the messages Men who did get circumcised after the intervention reported already contemplating it, and the conversation with their partner plus the transport voucher acted as a catalyst For the men who did not get circumcised, they and their partners cited well established barriers including lost wages, pain and religious/cultural reasons	Interventions using female partners are feasible but further work is needed to develop this	A pilot study with short follow‐up (three months), but was able to seek views of women, men and key informants
Downs (2017)	Focus groups with church leaders, nested in a community cluster randomized trial	Education of religious leaders who were then left to decide how to address circumcision in their community	Attitudes of religious leaders to male circumcision	There was considerable misinformation about and suspicion of circumcision among church leaders, and they would welcome more education (control villages) The intervention empowered church leaders and they reported high levels of acceptance among their communities (intervention villages) Church leaders recognized their strong influence felt they could be effective in promoting circumcision (all villages)	Working through religious leaders is an innovative model to promote healthy behaviour, addressing structural and cultural factors in a locally acceptable way	The large trial demonstrated impact. Focus groups with leaders but no qualitative data from participants

Bazant *et al*. and Evens *et al*. used focus group discussions and interviews to explore preferences for and perceptions of incentives 13, 15. While the lottery approach was reported to have promoted interest, “a buzz,” in the area, it was regarded by some as inappropriate (a smart phone), impractical (for people with no electricity) and the high value generated some suspicion 13. The other study suggested that the compensation removed a key barrier to uptake for some men but was of insufficient value for others 15.

Two studies used interviews and focus groups to explore perceptions of MTC and MTC+, football‐based interventions, one in relation to a trial and the other as part of a feasibility study in a new setting 14, 36. Younger participants were particularly appreciative of the coaches’ personal stories which helped allay fears, and their personal support was valued in both settings. In the feasibility study in Uganda it was felt that more attention should be paid to families and peers as well as coaches 36.

The role of influencers was further explored in two studies, one using pregnant women to target partners, the other aimed at religious leaders 18, 30. Both aimed to address social and family norms which can be barriers to uptake of VMMC. The pregnant women reported discomfort in delivering the message to partners, although together with the provision of travel vouchers it did seem to prompt some men who were already thinking about circumcision. In contrast the religious leaders seemed comfortable talking to their communities and recognized that their strong influence could be valuable.

Across the studies we identified the following emerging themes relevant to the success of demand creation interventions. First, interventions are better where they are judged appropriate and acceptable by the target population. Some responses to the smartphone lottery and of older men to MTC showed how a misjudged intervention may be counterproductive and raise suspicions and even undermine trust 13. Second, more personal interventions delivered by people with relevant experience can resonate well, as with the football interventions 14, 36. Third, addressing broader social and cultural influences through partnership with and education of community leaders can have a wide impact 30.

## 4 Discussion

### 4.1 Main findings

#### 4.1.1 Quantitative

The uptake or prevalence of VMMC increased with interventions from all four demand creation categories: financial, counselling, influencers and novel information delivery. The greatest population‐level impact (23% absolute increase in VMMC prevalence; 13,000 excess procedures; threefold relative increase in prevalence) was seen with a CRCT of a complex intervention that included a one‐day VMMC promotion training session for Church leaders 30. The largest relative increases in VMMC uptake (>4‐fold, absolute effect <8% increase) were seen with fixed financial incentives 19, 20, 31.

#### 4.1.2 Qualitative

We identified insights into how demand creation interventions based on addressing barriers can be more or less effective, for example financial incentives appeared more acceptable framed as fair compensation than as a lottery, but clearly have an important role particularly in “nudging” men who were already motivated or sensitized. Provision of information and support to overcome barriers related to knowledge of risks and benefits appeared acceptable when delivered by someone with direct personal experience or who was trusted and influential to the individual or group.

Taking both quantitative and qualitative evidence together, we consider that this study adds further insight into what works, how it works and why programmes need to be appropriate and acceptable in the particular setting and phase of the programme. This resonates with the growing interest in human‐based design approaches which adapt effective interventions to both the social context and individual preference.

### 4.2 Strengths and limitations

The main strength of this review is the comprehensive search strategy and broad inclusion criteria for qualitative and quantitative studies. This review extends the work of Tamuzi *et al*. as demand creation interventions of all types were assessed allowing comparisons between and within intervention types 23. The mixed methods approach builds on the work by Carrasco *et al*. and enhances understanding of contextual factors important to the interventions which should prove useful for policy makers 24.

The main limitation is that the reviewed studies were markedly heterogeneous. Data from seven of the fourteen priority SSA countries were included, and some interventions took place in rural and/or urban settings. Where the information was presented, there were large differences in baseline circumcision rates. The included studies used a range of study designs, measured different VMMC outcomes, reported varying effect measures and it was not possible to calculate the absolute impact of intervention in all studies.

The strength of the evidence from the included studies is variable; some used historic controls making it impossible to separate the impact of the interventions from secular changes; most had much shorter follow‐up than the anticipated two‐years needed for men to move from awareness to completing the procedure [37]; a number of studies were underpowered. With reference to the HIV Prevention Cascade, service capacity and other supply factors were outside the scope of this review but may have been limiting factors during some studies potentially hampering the impact of the intervention. In addition, intervention effectiveness was mostly viewed in isolation within the included studies, which may have neglected potential synergism within wider demand creation programmes.

Finally, no comprehensive search of the grey literature was conducted, and conference abstracts were excluded to allow quality assessment of the included studies which may have excluded recent studies in this rapidly evolving field.

### 4.3 Interpretation of results

This review adds to the growing body of evidence of the effectiveness of financial compensation at increasing VMMC uptake 23, 24. We found that higher compensation was associated with larger effect size; however, the observed absolute difference in uptake was small 31. It is important that the value of financial incentives in future programmes is optimized to maximize cost‐effectiveness. Carrasco *et al*. argue that it may be most cost‐effective to introduce financial incentives in settings with an already high prevalence of VMMC to focus on men who would not have “accessed the services otherwise” 24.

Although none of the included studies report major concerns with financial incentives, coercive potential should be monitored as qualitative data suggest this has the potential to impede programme effectiveness 13, 15. The type of compensation should be considered; food or transport vouchers may increase the amount that can be given without coercion fears or social harms 31; however, cash may have a higher perceived value and be more cost‐effective 13.

Individual counselling is effective at increasing VMMC uptake when offered to men who had already taken the step of presenting to a VMMC facility 33. Group education or awareness raising interventions delivered in the community are also effective 16, 28. Having time to process information between discussions allows for a natural decision‐making process; reviewing topics can reinforce benefits and alleviate new concerns 28. The design of counselling/education should also be considered in relation to the specific audience; group sessions may be more appropriate for younger men, who place greater value in peer acceptance and support particularly when delivered by people who had lived experience of VMMC 16, 25.

Theories have suggested that role models, families and peers play a part in changing an individual's behaviour 38. This review found that religious leaders could play a key role in disseminating information and appear to address the challenge of norms and social acceptability 30. Carrasco *et al*. found that a key barrier to men undergoing VMMC was a negative perception that it is “practiced by other or foreign cultures and religions” 25. The potential exists to work with other similarly influential community figures. A human‐centred design approach for new programmes would encourage open dialogue at the community level to identify appropriate influencers and their training needs 39.

VMMC peer‐referral schemes were not shown to increase VMMC uptake in this review. One key finding was that men did not want to refer their friends due to a reluctance to discuss potentially sensitive topics 21. We argue that further research into this intervention is warranted, using a human‐centred design approach, because we did not identify an appropriately powered RCT 22.

Studies across SSA, outside the scope of this review, have found that women can have a significant influence on decisions to become circumcised 25, 40, 41. The influence a woman has over the decision to undergo VMMC varies across culture, religion and marital status 40. Two studies in this review included women in the intervention 18, 29. Educating pregnant women about VMMC was unsuccessful at increasing demand; however, group education delivered to both partners was effective. Technology can increase the reach and reduce costs of demand creation programmes 17. This review found that the content of an SMS message is important as “encouraging” messages had a greater impact compared to basic health promotion 33.

### HIV prevention cascade and programme design

This review identified the need to undertake studies that are larger (to address the issues of underpowered research); have longer follow‐up (to allow for behavioural change); and that have consistent reporting of the impact of demand creation interventions. There is a need for consistency in outcome definitions, absolute and relative effects to allow more robust comparisons. Several of the studies included in this review did not report the observed absolute change in VMMC uptake and it is this absolute difference that is needed to populate the HIV Prevention Cascade to allow analysis of bottlenecks and inform the design of prevention programmes.

The impact of any lead‐time needed for behavioural change means that despite adequate supply of VMMC services, it will take time to see an impact on VMMC targets. Effective demand creation should disrupt this cycle, shortening the period considerably by increasing exposure to accurate information, addressing concerns and offering incentives.

The studies included in this review predominantly assess the effectiveness of single interventions. However, it is plausible that synergy (or redundancy) may occur when interventions are combined into demand creation programmes. Furthermore, the synergistic potential of VMMC demand creation strategies may extend to other areas of HIV prevention and healthcare. For example, Weiss *et al*. found that post‐intervention condom use increased after VMMC group education sessions, highlighting a potential for integration with sexual‐risk reduction services 28. We suggest that programmatic evaluations should draw on the HIV Prevention Cascade framework and consider applying a complex systems approach in recognition of the challenge of determining the contribution of individual interventions within the wider real‐world sphere of HIV prevention 8, 42.

## 5 Conclusions

A range of demand creation interventions can increase VMMC uptake, the most acceptable and effective interventions appear to be financial incentives framed as fair compensation and programmes of education or counselling delivered by people who are influential in the community or have personal experience of VMMC. In this context, individual interventions are often components of larger programmes which need to be appropriate and acceptable in the setting and phase of the programme. This resonates with the growing interest in human based design approaches which adapt effective interventions to both the social context and individual preference.

## Competing interests

The authors declare no competing interests.

## Authors’ contributions

H.W. conceived the study question and design. S.E. designed the systematic search and completed the initial search, data extraction and first draft as a student project under the supervision of H.W., T.R. and B.D. T.R. contributed to the qualitative analysis. B.D. undertook data extraction and quantitative analysis and co‐authored the revised drafts. H.W. undertook data extraction and qualitative analysis and co‐authored the revised draft. All authors edited and approved the final draft.

## Supporting information


**Appendix S1.** Inclusion criteria.
**Appendix S2.** Example search strategy for Medline.
**Appendix S3.** Exclusion criteria.
**Appendix S4.** Questions and results of MMAT.
