# Supplementary material for: The effectiveness of demand creation interventions for voluntary male medical circumcision for HIV prevention in sub‐Saharan Africa: a mixed methods systematic review
Source: J Int AIDS Soc. 2019 Jul 22;22(Suppl Suppl 4):e25299. doi: 10.1002/jia2.25299 (PMC6643070; doi:10.1002/jia2.25299)
Supplement: Supplementary file 1 — Appendix S1. Inclusion criteria. Appendix S2. Example search strategy for Medline. Appendix S3. Exclusion criteria. Appendix S4. Questions and results of MMAT. [file JIA2-22-e25299-s001.docx]

**Appendix A. Inclusion criteria.**

| Population | Men in Sub-Saharan Africa at least 10 years old |
| --- | --- |
| Intervention | Strategy to generate demand for Voluntary Male Medical Circumcision (VMMC) services |
| Comparison | Usual standard of care in area or no service |
| Outcome | Qualitative or quantitative measure of uptake or prevalence of VMMC |
| Study design | RCTs, quasi-experimental, case-control, cohort, comparative and observational |

**Appendix B. Example search strategy for Medline.**

1. Circumcision, Male/
2. Circumcision or circumcis* or uncircumcis* or VMMC or MMC
3. 1 or 2
4. Demand* or uptake or interest* or agree* or ?ndorse* or accept* or support or sponsor or encourage or promot* or enable or empower or permit or prevent or obstacle or access* or facilitate* or incentive* or factor* reason* or practice* or adopt* or accept* or utili?*
5. exp HIV/
6. hiv or hiv-1* or hiv-2* or hiv1 or hiv2 or human immunodeficiency virus or human immunedeficiency virus or human immuno-deficiency virus or human immune-deficiency virus or (human immun* and deficiency virus) or acquired immunodeficiency syndrome or acquired immunedeficiency syndrome or acquired immuno-deficiency syndrome or acquired immune-deficiency syndrome or (acquired immun* and deficiency syndrome)
7. 4 or 5
8. exp "Africa South of the Sahara"/
9. (Africa* adj4 (Sub?Sahara* OR Subsahara* OR South of the Sahara*)
10. (Africa* adj4 (East* OR West* OR Mid* OR Centr* OR South*)
11. Black Africa*
12. British Indian Ocean Territory or Angola or B?nin or Botswana or Burkina Faso or Burundi or Cabo Verde or Cape Verde or Camero?n or Central African Republic or CAR or Republique centrafricaine or Centrafrique or Chad or Republique du Tchad or Comor?s or Komori or Congo or Congo-Brazzaville or Cote d?Ivoire or Ivory Coast or DRC or DROC or Congo-Kinshasa or Zaire or Djibouti or Equatorial Guinea or Guinea Ecuatorial or Guinee equatorial or Guine Equatorial or Eritrea or Ethiopia or French Southern 'and' Antarctic or TAAF or Gabon* or Republique gabonaise or Gambia or Ghana or Guinea or Republique de Guinee or Guinea-Bissau or Republica da Guine-Bissau or Kenya or Lesotho or Liberia or Madagascar or Madagasikara or Malawi or Mali or Mauritania or Mauritius or Republique de Maurice or Mayotte or Mo?ambique or Namibi? or Niger or Nigeria or Reunion or Rwanda or S?o Tom? 'and' Pr?ncipe or Sao Tome e Principe or Saint Helena or S?n?gal or Seychelles or Sesel or Sierra Leone or Somalia or South Africa or RSA or South Sudan or Swaziland or kaNgwane or Togo* or Uganda or Tanzania or Zambia or Zimbabwe
13. 6 or 7 or 8 or 9 or 10
14. clinical trial/ or observational study/ or comparative study/
15. comparative* OR comparison* OR evaluat* OR follow-up stud* OR control* OR prospectiv* OR retrospectiv* OR volunteer* OR randomi* OR non-randomi* OR before after OR time series OR case-control OR cross-section* OR longitud* OR descripti* OR evaluat* or quasi experiment*
16. 14 or 15
17. 3 and 4 and 7 and 13 and 16

**Appendix C. Exclusion criteria.**

| Study population not in Sub-Saharan Africa |
| --- |
| Study population only includes infants |
| Not concerned with VMMC for HIV prevention |
| Study not concerned with increasing delivery of VMMC |
| No measure of data concerned with changes in uptake |
| Intervention designed to increase supply instead of demand |

**Appendix D. Questions and results of MMAT.**

| Screening questions | 1. Are there clear qualitative and quantitative research questions (or objectives*), or a clear mixed methods question (or objective*)? |
| --- | --- |
|  | 2. Do the collected data allow address the research question (objective)? E.g., consider whether the follow-up period is long enough for the outcome to occur (for longitudinal studies or study components). |
| 1. Qualitative | 1.1. Are the sources of qualitative data (archives, documents, informants, observations) relevant to address the research question (objective)? |
|  | 1.2. Is the process for analysing qualitative data relevant to address the research question (objective)? |
|  | 1.3. Is appropriate consideration given to how findings relate to the context, e.g., the setting, in which the data were collected? |
|  | 1.4. Is appropriate consideration given to how findings relate to researchers’ influence, e.g., through their interactions with participants? |
| 2. Quantitative randomized controlled (trials) | 2.1. Is there a clear description of the randomization (or an appropriate sequence generation)? |
|  | 2.2. Is there a clear description of the allocation concealment (or blinding when applicable)? |
|  | 2.3. Are there complete outcome data (80% or above)? |
|  | 2.4. Is there low withdrawal/drop-out (below 20%)? |
| 3. Quantitative nonrandomized | 3.1. Are participants (organizations) recruited in a way that minimizes selection bias? |
|  | 3.2. Are measurements appropriate (clear origin, or validity known, or standard instrument; and absence of contamination between groups when appropriate) regarding the exposure/intervention and outcomes? |
|  | 3.3. In the groups being compared (exposed vs. non-exposed; with intervention vs. without; cases vs. controls), are the participants comparable, or do researchers take into account (control for) the difference between these groups? |
|  | 3.4. Are there complete outcome data (80% or above), and, when applicable, an acceptable response rate (60% or above), or an acceptable follow-up rate for cohort studies (depending on the duration of follow-up)? |
| 4. Quantitative descriptive | 4.1. Is the sampling strategy relevant to address the quantitative research question (quantitative aspect of the mixed methods question)? |
|  | 4.2. Is the sample representative of the population understudy? |
|  | 4.3. Are measurements appropriate (clear origin, or validity known, or standard instrument)? |
|  | 4.4. Is there an acceptable response rate (60% or above)? |
| 5. Mixed methods | 5.1. Is the mixed methods research design relevant to address the qualitative and quantitative research questions (or objectives), or the qualitative and quantitative aspects of the mixed methods question (or objective)? |
|  | 5.2. Is the integration of qualitative and quantitative data (or results*) relevant to address the research question (objective)? |
|  | 5.3. Is appropriate consideration given to the limitations associated with this integration, e.g., the divergence of qualitative and quantitative data (or results*) in a triangulation design? |

| **Autor (Date)** | **Study type** | **Screening questions** | **1** | **2** | **3** | **4** | **Final score** |
| --- | --- | --- | --- | --- | --- | --- | --- |
| Barnabas (2016) | Quantitative (RCT) | Y/Y | Y | Y | Y | Y | 100% |
| Bazant (2016) | Quantitative (RCT) | Y/Y | Y | N | Y | N | 50% |
|  | Qualitative |  | Y | Y | Y | N |  |
|  | Mixed |  | Y | Y | N |  |  |
| Cook (2016) | Quantitative (RCT) | Y/Y | Y | Y | Y | Y | 100% |
| DeCelles (2016) | Qualitative | Y/Y | Y | Y | Y | N | 75% |
| Downs (2017) | Quantitative (RCT) | Y/Y | Y | Y | Y | N/A | 75% |
|  | Qualitative |  | Y | Y | Y | N |  |
|  | Mixed |  | Y | Y | N |  |  |
| Evens (2016) | Qualitative | Y/Y | Y | Y | Y | N | 75% |
| Kaufman (2016) | Quantitative (RCT) | Y/Y | Y | Y | Y | N | 75% |
| Leiby (2016) | Quantitative (RCT) | Y/Y | N | N | N | Y | 25% |
| Marshall (2017) | Quantitative (Non-random) | Y/Y | Y | Y | Y | Y | 100% |
| Montague (2014) | Quantitative (Non-random) | Y/Y | Y | Y | Y | N | 75% |
| Miiro (2017) | Quantitative (Non-random) | Y/Y | Y | Y | N | Y | 75% |
|  | Qualitative |  | Y | Y | Y | N |  |
|  | Mixed |  | Y | Y | N |  |  |
| Semeere (2016) | Quantitative (Non-random) | Y/Y | Y | Y | Y | N | 75% |
|  | Qualitative |  | Y | Y | Y | N |  |
|  | Mixed |  | Y | Y | N |  |  |
| Thirumurthy (2014) | Quantitative (RCT) | Y/Y | Y | Y | Y | Y | 100% |
| Thirumurthy (2016) | Quantitative (RCT) | Y/Y | Y | Y | Y | Y | 100% |
| Thornton (2016) | Quantitative (RCT) | Y/Y | N | N | Y | Y | 50% |
| Weiss (2015) | Quantitative (RCT) | Y/Y | Y | Y | Y | Y | 100% |
| Wilson (2016) | Quantitative (RCT) | Y/Y | Y | Y | Y | N/A | 75% |
| Zanolini (2016) | Quantitative (Non-random) | Y/Y | Y | Y | Y | Y | 100% |
| Y=yes; N=No; N/A= Not applicable | | | | | | | |
